# Staging the axilla in women with breast cancer: the utility of preoperative ultrasound-guided needle biopsy

**DOI:** 10.7497/j.issn.2095-3941.2014.02.001

**Published:** 2014-06

**Authors:** Nehmat Houssami, Robin M. Turner

**Affiliations:** Screening and Test Evaluation Program (STEP), School of Public Health, Sydney Medical School, University of Sydney, Sydney 2006, Australia

**Keywords:** Breast cancer, axillary staging, node metastases, test utility, ultrasound-guided needle biopsy (UNB)

## Abstract

Preoperative staging of the axilla in women with invasive breast cancer using ultrasound-guided needle biopsy (UNB) identifies approximately 50% of patients with axillary nodal metastases prior to surgical intervention. Although moderately sensitive, it is a highly specific staging strategy that is rarely falsely-positive, hence a positive UNB allows patients to be triaged to axillary lymph-node dissection (ALND) avoiding potentially unnecessary sentinel node biopsy (SNB). In this review, we extend our previous work through an updated literature search, focusing on studies that report data on UNB utility. Based on data for 10,934 breast cancer patients, sourced from 35 studies, a positive UNB allowed triage of 1,745 cases (simple proportion 16%) to axillary surgical treatment: the utility of UNB was a median 19.8% [interquartile range (IQR) 11.6%-26.7%] across these studies. We also modelled data from a subgroup of studies, and estimated that amongst patients with metastases to axillary nodes, the odds ratio (OR) for high nodal disease burden for a positive UNB versus a negative UNB was 4.38 [95% confidence interval (95% CI): 3.13, 6.13], *P*<0.001. From this model, the estimated proportion with high nodal disease burden was 58.9% (95% CI: 50.2%, 67.0%) for a positive UNB, whereas the estimated proportion with high nodal disease burden was 24.6% (95% CI: 17.7%, 33.2%) if UNB was negative. Overall, axillary UNB has good clinical utility and a positive UNB can effectively triage to ALND. However, the evolving landscape of axillary surgical treatment means that UNB will have relatively less utility where surgeons have modified their practice to omission of ALND for minimal nodal metastatic disease.

## Introduction

Surgical management of the axilla in women with invasive breast cancer has changed considerably in the last two decades: sentinel node biopsy (SNB) has replaced axillary lymph node dissection (ALND) as the primary surgical staging approach^1^^,^^2^ with selective ALND based on the status of the sentinel node(s). More recently, evidence from a landmark randomized trial (Z0011)^3^ has shown that omission of ALND may be appropriate in defined groups of patients (clinical stage T_1-2_ and N_0_ patients having breast conservation and whole-breast radiation) with minimal sentinel node disease burden. It is not surprising then that the evolution of surgical treatment of the axilla has shaped the role of preoperative staging, specifically the use of preoperative axillary ultrasound with selective ultrasound-guided needle biopsy (UNB). In this review, we estimate and discuss the utility of UNB highlighting the role of preoperative UNB and its consequences on surgical management of the axilla.

## Background on clinical utility of a test

Test accuracy describes the ability of a test to rule in or out a disease or to assess disease severity. On the other hand clinical utility of a test represents the capacity to use the information from the test to enable a decision to adopt, or to reject, a therapy or an intervention. Test utility expresses^4^ to what extent testing contributes to improving health outcomes. Bossuyt *et al*.^4^ report that key features of test clinical utility are that use of the test improves health outcomes, and that the test forms part of a strategy whereby health outcomes are generated ‘not only by using the test but also by a management strategy that starts with testing but includes all downstream consequences of subsequent clinical management’^4^. In the specific scenario of the axilla in invasive breast cancer, knowledge of the status of axillary nodes prior to surgical intervention can affect treatment planning. Two meta-analyses^5^^,^^6^, each based on a large number of original studies and hence large datasets, have reported that a preoperative strategy of ultrasound with selective UNB of abnormal-appearing or suspicious axillary node(s) correctly identifies approximately 50% of breast cancer patients who have node metastases. Diepstraten *et al*.^6^ estimated this strategy to have a sensitivity of 50% [95% confidence interval (95% CI): 43%, 57%] and Houssami *et al*.^5^ reported it as a median 55.2% (IQR 41.8%-68.2%) across primary studies. On this basis exists the potential utility of preoperative UNB whereby ultrasound-directed needle biopsy can confirm metastases to the axillary nodes, enabling triage to ALND and a single axillary operation.

## Background on ultrasound accuracy

Using ultrasound with selective UNB (based on ultrasound features of nodes) for preoperative staging of the axilla in newly diagnosed breast cancer patients has been practiced for many years^5^^,^^7^^,^^8^, however it is noteworthy that the progressive use of this approach is not solely related to accuracy. It has been partly due to the relative efficiency and modest cost of this combination strategy, the long-established use of ultrasound in breast diagnosis, and because breast ultrasound is a patient-friendly form of image-guided intervention. It should also be noted that ultrasound on its own yields moderate and variable accuracy: meta-analysis^5^ of data (4,313 subjects) from 21 studies^8^^-^^28^ found a median ultrasound sensitivity of 61.4% (IQR 51.2%-79.4%) and a median ultrasound specificity of 82.0% (IQR 76.9%-89.0%). Therefore the addition of UNB (directed by axillary ultrasound features) is intended to improve both the sensitivity and the specificity of preoperative staging, and in particular to substantially improve its specificity such that a positive UNB can be used to plan surgical treatment of the axilla. So, using data from the same meta-analysis, it can be shown that for the subset of 1,733 patients who were selected to UNB, the median UNB sensitivity is 79.4% (IQR 68.3%-88.9%) and the median UNB specificity is 100% (IQR 100%-100%)^5^. Various criteria have been used to define abnormal nodes, including morphologic features and/or node size (enlarged nodes), and to select patients to UNB; some of the most frequently reported morphologic features^12^^,^^13^^,^^16^^,^^17^^,^^24^^,^^29^^-^^31^ defining suspicious nodes are:

- thickening of the cortex (primary studies have used various thresholds to define thickening, usually 2-3 mm, but some studies have used a wider mm threshold to define thickening); cortical thickening may be diffuse or focal;
- cortex shape/appearance: eccentric or irregular; asymmetric; lobulated (uni- or multi-lobulation);
- absence/loss of central fatty hilum (this criterion is predictive of metastases but it is not frequently present so may be insensitive);
- rounded nodes (ratio of the longitudinal and transverse dimensions).

## Review methods

We previously reported a systematic evidence review on the accuracy and utility of UNB^5^. In the present review, we extend our previous work focusing on studies that report data on UNB utility. Because the accuracy of ultrasound and UNB has been comprehensively reported in our meta-analysis^5^ and also in another more recent meta-analysis from Diepstraten *et al*.^6^, we will not repeat analyses of ultrasound and UNB accuracy. Instead, we present a summary table of findings from these previous meta-analyses (**Table 1**) to inform readers of reported accuracy estimates. For the present analysis, we extended our previous review by updating the literature search strategy described by Houssami *et al*.^5^, and performed this at January 2014 (Medline and Pre-Medline search). Studies were eligible for the updated review if they provided data on ultrasound-guided fine needle aspiration biopsy (FNAB) or core needle biopsy (CNB) of axillary nodes (collectively referred to as UNB) in women with invasive breast cancer, and if they provided data that quantify or allow estimation of clinical utility. We defined utility as the proportion of women triaged to axillary surgery or axillary treatment as previously defined^5^. Amongst all eligible studies (from the previous and the updated search) we also looked for data that would allow investigation of UNB results in relation to nodal disease burden.

**Table 1:** Accuracy of preoperative ultrasound & UNB for staging the axilla in invasive breast cancer based on two meta-analyses

Measures of accuracy or utility [number in analysis]	Summary statistic or estimate (95% CI or IQR)^§^ (%)
Accuracy	
Ultrasound alone [4,313]^5^	Median sensitivity: 61.4 (IQR, 51.2-79.4); Median specificity: 82.0 (IQR, 76.9-89.0)
Ultrasound +/– UNB [9,212]^6^	Pooled sensitivity: 50.0 (CI, 43.0, 57.0)
Ultrasound +/– UNB [9,212]^6^	False negative rate*: 25 (CI, 24, 27)
Cases selected to UNB: UNB accuracy [2,805, excludes insufficient results]^5^	Pooled sensitivity: 79.6^†^ (CI, 74.1, 84.2) Pooled specificity: 98.3 (CI, 97.2, 99.0)
Cases selected to UNB: UNB predictive values [2,874]^5^	Median PPV: 100 (IQR, 100-100) Median NPV: 67.4 (IQR, 60.0-76.2)

^§^, 95% CI given for modeled (pooled) estimates, IQR given for median proportion of summarized data; *, Estimated proportion of women with a negative ultrasound +/– UNB (ultrasound with selective UNB) who are found to have axillary nodal metastases at SNB; ^†^, Meta-analysis^5^ estimated the sensitivity of axillary UNB (for all breast cancer patients who had UNB) as 79.6% based on thirty studies, however recent work which confirms a similar sensitivity of 79% for all breast cancer patients also shows that UNB sensitivity of axillary nodes is much lower (33%) in the subgroup with invasive lobular histology^32^. UNB, ultrasound-guided needle biopsy; IQR, inter-quartile range; SNB, sentinel node biopsy; UNB, ultrasound-guided needle biopsy.

## Statistical analysis

Descriptive statistics (median and IQR) were used to describe UNB utility, which was calculated as the simple proportion of women triaged to axillary surgery or axillary treatment, from all subjects included in the study (therefore the denominator for this calculation was not restricted to women who had UNB but included all cases). Because we previously found evidence of a positive linear correlation between UNB utility and the underlying prevalence (study-specific proportion) of axillary node metastases across studies, we also calculated descriptive statistics for underlying prevalence of axillary node metastases. We used a bubble plot to demonstrate the relationship between UNB utility and the underlying prevalence of axillary node metastases across all studies.

For the subset of studies that provided data on UNB results in relation to node disease burden, we used logistic regression modelling incorporating a random-effect for study to investigate nodal disease burden according to whether UNB was positive versus negative. Node disease burden was examined in the model by analysing the proportion of patients with high nodal disease burden (defined as >3 metastatic nodes in the majority of studies) from all patients with axillary node metastases (total of low and high node disease burden), by UNB result. Therefore the model estimated the odds ratio (OR) and corresponding 95% CI for high nodal disease burden in patients with a positive UNB versus those with a negative UNB.

## Results

**Figure 1:**
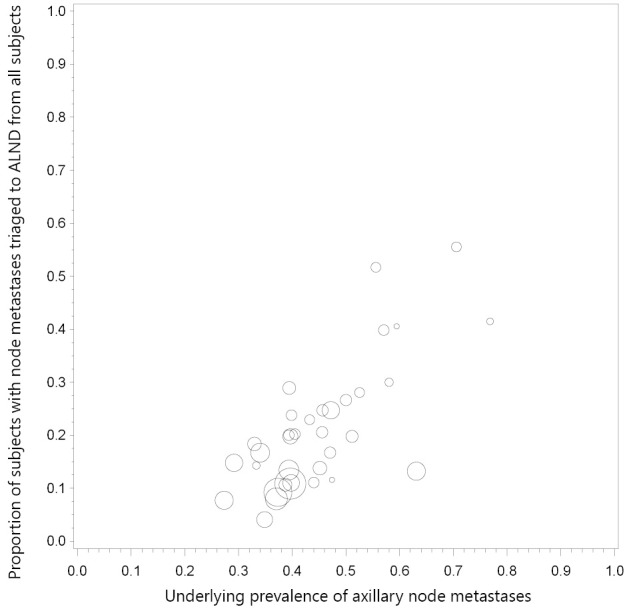
Bubble plot shows study-specific UNB utility (proportion of subjects triaged to axillary surgery based on UNB result) in relation to underlying prevalence of node metastases.

Our updated search yielded 35 eligible studies^9^^,^^11^^-^^17^^,^^21^^-^^23^^,^^25^^-^^28^^,^^30^^,^^31^^,^^33^^-^^50^ providing data on 10,934 patients with breast cancer in whom a positive UNB result allowed triage of 1,745 cases (simple proportion 16%) to axillary surgical treatment: the utility of UNB was a median 19.8% (IQR 11.6%-26.7%) across all studies^9^^,^^11^^-^^17^^,^^21^^-^^23^^,^^25^^-^^28^^,^^30^^,^^31^^,^^33^^-^^50^. Axillary treatment consisted of triage directly to ALND for the vast majority (and avoidance of SNB) but in some studies UNB was used to affect neoadjuvant therapy prior to ALND^9^^,^^31^^,^^41^^,^^47^. The median prevalence of node metastases (proportion of patients found to have node metastases on surgical histology) across the 35 studies was 43.2% (IQR 38.7%-51.2%)^9^^,^^11^^-^^17^^,^^21^^-^^23^^,^^25^^-^^28^^,^^30^^,^^31^^,^^33^^-^^50^. In **Figure 1**, the bubble plot (bubble size reflects study size) displays study-specific proportion of utility (proportion of subjects triaged to axillary surgery based on UNB result) in relation to study-specific underlying prevalence of node metastases.

**Figure 2:**
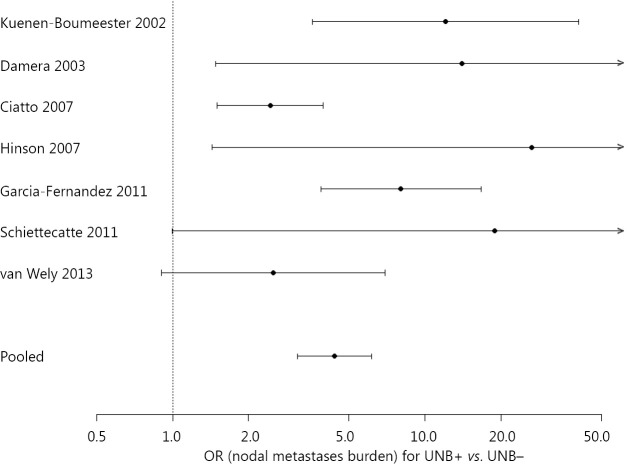
Estimated OR for high nodal disease burden in patients with a positive UNB *vs*. those with a negative UNB amongst patients with axillary node metastases. Study-specific and pooled estimates shown in plot; pooled OR=4.38 (95% CI: 3.13, 6.13); a positive UNB refers to positive ultrasound/positive needle biopsy, a negative UNB refers to positive ultrasound/negative needle biopsy amongst patients subsequently shown to have nodal metastases on SNB and/or ALND; high nodal disease burden refers to >3 nodes (relative to 1-3 nodes) however the study from Garcia-Fernandez 2011 used ≥2 nodes to classify higher node disease burden. UNB, ultrasound-guided needle biopsy; SNB, sentinel node biopsy; OR, odds ratio; ALND, axillary lymph-node dissection.

Seven studies^20^^,^^34^^,^^35^^,^^38^^,^^42^^,^^43^^,^^48^ contributed to the model that estimated the OR for high nodal disease burden in patients with a positive UNB versus those with a negative UNB: study-specific and pooled estimates are shown in **Figure 2**. Based on the model, the OR for high nodal disease burden for a positive UNB (versus negative UNB) was 4.38 (95% CI: 3.13, 6.13), *P*<0.001. The estimated proportion of patients with high nodal disease burden (>3 nodes) from this model was 58.9% (95% CI: 50.2%, 67.0%) for a positive UNB result, whereas the estimated proportion with high nodal disease burden was 24.6% (95% CI: 17.7%, 33.2%) if UNB was negative.

## Discussion

In this review, we summarize the clinical utility of axillary UNB as a median 19.8% (IQR 11.6%-26.7%) across 35 primary studies, based on 10,934 patients with breast cancer in whom a positive UNB result allowed triage to axillary surgical treatment (and hence avoidance of two-stage axillary surgery) for those harbouring nodal metastases. This median proportion has been calculated from all subjects included in these studies, therefore it also includes all those who had axillary ultrasound but did not proceed to needle biopsy. The UNB utility % is essentially unchanged from our earlier meta-analysis which reported a utility ranging between 17.7% and 19.8% (based on data for 4,941 patients) representing the proportion of all subjects who are triaged, or could be triaged, directly to ALND^5^ thus avoiding SNB. The UNB utility proportions we describe represent good clinical utility in the context of a management framework whereby patients with UNB-confirmed axillary node metastases can proceed to ALND given the high specificity of the test (and assuming at least moderate sensitivity as estimated in meta-analyses^5^^,^^6^). However, when interpreting UNB utility, it should be noted that many of the studies had moderate to high underlying prevalence of node metastases (**Figure 1**) implying that published studies may have selected patients at relatively higher risk of harbouring metastatic nodes, which could over-estimate UNB utility.

Another utility for preoperative ultrasound and UNB, described in several studies, relates to axillary staging in patients who are to receive neoadjuvant therapy^9^^,^^13^^,^^22^^,^^24^^,^^26^^,^^31^^,^^47^^,^^51^^,^^52^. For example, one study^52^ reported that UNB for axillary staging can support planning of neoadjuvant therapy in breast cancer patients, and other studies included in our analysis reported use of this axillary staging strategy to affect neoadjuvant therapy followed by axillary surgery^9^^,^^31^^,^^41^^,^^47^.

In this review, we considered studies that provided data on ultrasound-guided FNAB or CNB of axillary nodes, which we collectively referred to as UNB; however, clinicians may want to know whether there are differences in the accuracy between FNAB and CNB for preoperative axillary staging. In our earlier work we compared sensitivity for studies using FNAB with those using CNB but we did not find statistically significant differences^5^—this is most likely because the vast majority of published studies used FNAB, or used FNAB or CNB in the same study but most subjects had FNAB, which limits meaningful statistical comparisons. Of note, when we examined data in our previous meta-analysis and included insufficient UNB (as negative test result), we found some difference in sensitivity of FNAB (72.2%; 95% CI: 63.9%, 79.3%) and CNB (83.3%; 95% CI: 70.0%, 91.4%) than estimates that excluded insufficient data from the analysis, but this difference between FNAC and CNB sensitivity was not statistically significant (*P*=0.13)^5^. Therefore, either FNAB or CNB of the axilla provide good accuracy in this clinical context and can be used to manage patients. In the updated literature review, we identified one study that uniquely performed both FNAB and CNB on the (same) suspicious node in each of 66 patients, meaning that each suspicious axillary node was sampled within-patient using both FNAB and CNB. In this study, Rautiainen *et al*.^30^ reported that the sensitivities of FNAB and CNB were 72.5% and 88.2% respectively (*P*=0.008), and specificity was 100% for FNAB and for CNB (as was PPV). Although this was a relatively small study, it showed that CNB is more sensitive than FNAB, however, each of these tests are highly specific in the preoperative axillary staging setting and hence either of these axillary UNB tests would confer good utility for triaging to axillary surgery.

Because the accuracy of UNB has been comprehensively reported in our meta-analysis^5^ and also in another recent meta-analysis from Diepstraten *et al*.^6^, we did not repeat analyses of UNB accuracy in this paper, instead we summarized this information (**Table 1**). However, it was noted through our updated literature search that some studies reported data showing that UNB is much more likely to be correctly positive(more sensitive) if macro metastases are present in the axillary nodes than if only micrometastases (<2 mm) are present^16^^,^^31^^,^^43^^,^^49^. Britton *et al*.^16^ reported UNB sensitivity as 60.3% for macro metastases and as <30% for micrometastases; and Garcia-Ortega^31^ reported a sensitivity of UNB of 71% for macro metastases whereas none of 12 cases (0%) with micrometastases were correctly diagnosed using UNB.

We extended our previous work to describe UNB utility as the proportion of women (from all subjects) triaged to ALND, based on published studies at January 2014. It is noteworthy that we had also examined a related measure of UNB utility in our earlier review^5^, defined as the median proportion of women with metastatic axillary nodes triaged or potentially triaged directly to ALND if UNB is used routinely in patients with invasive breast cancer: the previous analysis was based on 2,162 UNBs in 4,451 subjects, and showed that UNB utility measure differed by (study-level) median tumor size^5^. We reported that the median proportion of women with metastatic axillary nodes triaged was 42.2% (IQR 30.6%-49.2%) for studies with a median tumor size <21 mm, and 65.6% (IQR 48.9%-69.7%) for studies with median tumor size ≥21 mm, and the OR for the proportion triaged in median tumor size ≥21 mm relative to <21 mm studies was 2.57 (95% CI: 1.29, 5.09), *P*=0.0095. This indicates that axillary UNB has significantly higher utility in women with larger cancers, which is not surprising given that they have a relatively higher likelihood of having nodal metastases than women with smaller cancers.

While the above data and discussion to this point highlight the clinical utility of axillary UNB, given the evidence from the Z0011 trial^3^^,^^53^ and its apparent impact on practice^54^, raises the issue of whether UNB remains useful at present and whether it will have utility in breast cancer staging in the future. In the subgroup of patients defined by the Z0011 criteria (clinical stage T_1-2_ and N_0_ patients having breast conservation and whole-breast radiation)^3^^,^^53^ there may be relatively less utility for UNB because patients with node metastases in only 1-2 sentinel nodes would not necessarily be managed with ALND: hence the utility of axillary ultrasound with UNB will depend on whether or not the surgeon has adopted omission of ALND in patients with minimal sentinel node disease. Surgeons who have modified their practice according to the Z0011 trial^3^^,^^53^ may find preoperative axillary ultrasound with UNB of limited or questionable utility, because there is little evidence that axillary UNB can differentiate between minimal and more advanced nodal disease.

We therefore interrogated existing data on UNB in an attempt to gain further insights regarding UNB outcome and nodal disease burden. However, only seven studies identified in our review^20^^,^^34^^,^^35^^,^^38^^,^^42^^,^^43^^,^^48^ provided relevant information allowing us to model data in a subgroup analysis—we estimated that amongst patients with axillary nodal metastases, the OR for high nodal disease burden in those with a positive UNB versus those with a negative UNB was 4.38 (95% CI: 3.13, 6.13), *P*<0.001. This means that the odds of harbouring high nodal disease burden are significantly increased in patients who have a positive UNB relative to those who have a negative UNB. From this model, the estimated proportion of patients with high nodal disease burden was 58.9% for a positive UNB, whereas the estimated proportion with high nodal disease burden was 24.6% if UNB was negative. These findings indicate that amongst those with a positive UNB there is a relatively higher proportion of patients with high nodal disease burden but still a substantial proportion (approximately 40%) will have low nodal disease burden. So overall, axillary UNB has good clinical utility and a positive UNB can effectively triage to ALND. However, the changing landscape of axillary surgical treatment means that a positive UNB may have relatively less utility where surgeons have modified their practice to omission of ALND for minimal nodal metastatic disease. Also, our model is limited by the paucity of studies contributing data to this subgroup analysis.

Although the utility of UNB appears questionable if there is broader and progressive adoption of SNB-only for minimal axillary node metastases, it is possible that the reverse may occur. Because the algorithm for axillary surgical management in invasive breast cancer is evolving, this could result in a potentially more pragmatic approach to the application of axillary ultrasound with UNB. For example, axillary ultrasound might be used to look for multiple abnormal nodes, and to triage those with multiple metastatic nodes to ALND. Other more novel possibilities include enhanced application of ultrasound, either through refined systematic scanning of the axilla (as shown by Britton *et al*.^55^), or through technologic developments, for example contrast-enhanced ultrasound^56^^,^^57^, mayallow precise UNB-sampling of the sentinel node(s). Britton and colleagues have described systematic scanning of the axilla, with emphasis on level I nodes and with particular attention to identifying the lowest 1-2 nodes, and have reported that use of that approach can lead to UNB of sentinel nodes in 64% of patients^55^. Because false negative ultrasound and UNB may be due to failure to find and sample the sentinel node, or may be due to failure to adequately sample metastatic disease within correctly identified diseased sentinel node(s), Sever *et al*. have investigated contrast-enhanced ultrasound with microbubbles (injected intradermally in the periareolar region)^56^^,^^57^: this research showed the potential to improve identification as well as sampling of the sentinel node(s) through targeted UNB of the microbubble enhancing axillary lymph node^57^. Such novel approaches to preoperative axillary staging, that support identification and/or sampling of sentinel nodes, could mean that many patients may not require any axillary intervention in future (in the context of the results of Z0011 trial^3^^,^^53^). So further research to develop and evaluate these staging strategies would be worthy and could contribute to future practice.

An important study in this field is a trial currently in progress in Europe: a prospective randomized controlled trial using axillary ultrasound to decide surgical management of the axilla [Sentinel node *vs*. Observation after axillary Ultra-Sound (SOUND) trial] in patients with early breast cancer (tumors ≤2 cm and clinically node-negative axillae) who are candidates for breast-conserving surgery^58^. Patients will have axillary ultrasound to assess whether or not they have suspicious nodal involvement, and those shown to have a negative ultrasound or (for a single abnormal node) negative UNB will be randomized to SNB or no further axillary surgery. The SOUND trial represents yet another possibility for a potential shift in axillary management towards less intervention and may see an extended role for axillary ultrasound with selective UNB in breast cancer staging in future^58^.

## Conclusion

Preoperative ultrasound-based staging of the axilla using ultrasound with selective UNB is moderately sensitive but highly specific and provides a staging strategy that allows patients to be triaged to ALND (based on a positive result); this helps avoid unnecessary two-stage axillary surgery, whereas those with a negative UNB proceed to standard SNB for staging. A large number of non-randomised studies report that UNB provides good clinical utility for axillary surgical management, quantified in this paper as a median utility of 19.8% (IQR 11.6%-26.7%) of breast cancer patients (across 35 studies) who can be triaged to axillary surgery based on a positive UNB, and without SNB. However, ongoing evolution of axillary surgical treatment may render preoperative axillary UNB less useful depending on local surgical practice, and specifically on whether omission of ALND in patients with minimal nodal metastatic burden has been adopted into practice. Future research that allows enhanced application of ultrasound with UNB to identify and target sentinel node(s) and/or to discriminate between minimal versus advanced axillary nodal metastatic involvement is likely to contribute substantially towards management of the axilla in invasive breast cancer.
